# Cardiovascular Protection in Coronary Artery Disease: Mechanistic and Clinical Insights into SGLT2 Inhibitors and GLP-1 Receptor Agonists

**DOI:** 10.3390/ph18081202

**Published:** 2025-08-14

**Authors:** Nicola Cosentino, Filippo Trombara, Monica De Metrio, Chiara Molinari, Stefano Genovese, Gianluca Pontone, Giancarlo Marenzi

**Affiliations:** 1 Centro Cardiologico Monzino IRCCS, Via Parea 4, 20138 Milan, Italy; filippo.trombara@cardiologicomonzino.it (F.T.); monica.demetrio@cardiologicomonzino.it (M.D.M.); chiara.molinari@cardiologicomonzino.it (C.M.); stefano.genovese@cardiologicomonzino.it (S.G.); gianluca.pontone@cardiologicomonzino.it (G.P.); giancarlo.marenzi@cardiologicomonzino.it (G.M.); 2 Department of Biomedical, Surgical and Dental Sciences, University of Milan, 20133 Milan, Italy

**Keywords:** coronary artery disease, acute myocardial infarction, sodium-glucose cotransporter-2 inhibitors, glucagon-like peptide-1 receptor agonists

## Abstract

Coronary artery disease (CAD) and its acute manifestation, myocardial infarction (AMI), remain significant global health burdens, with a substantial impact on morbidity and mortality, especially in individuals with Type 2 Diabetes Mellitus (T2DM). The interaction between hyperglycemia, endothelial dysfunction, inflammation, and thrombosis creates a complex pathological environment that contributes to the progression of CAD and its acute complications, including AMI. Over recent years, there has been a shift in the therapeutic approach to CAD, especially in T2DM patients, where pharmacologic agents originally developed for glycemic control have demonstrated notable cardiovascular benefits beyond glucose regulation. Sodium-glucose cotransporter-2 inhibitors (SGLT2i) and glucagon-like peptide-1 receptor agonists (GLP-1 RAs) are at the forefront of this paradigm shift. Initially, these agents were designed to improve glycemic control, but their broader cardiovascular protective effects have become increasingly evident, particularly in patients with CAD. This review aims to provide an in-depth exploration of the mechanistic underpinnings of these agents, the clinical data supporting their cardiovascular benefits, and their potential role for patients with CAD.

## 1. Introduction

Coronary artery disease (CAD) is a progressive condition characterized by endothelial dysfunction, the accumulation of lipid-laden plaques within the coronary arteries—leading to lumen narrowing and reduced blood flow—and the potential for plaque rupture [1,2]. The clinical consequence of these processes is acute myocardial infarction (AMI), which continues to be a major cause of morbidity and mortality worldwide [2,3]. Individuals with diabetes mellitus (DM) face a disproportionate burden of CAD due to the exacerbating effects of chronic hyperglycaemia, systemic inflammation, and accelerated atherogenesis [4,5]. Conventional strategies for managing CAD, including antiplatelet agents, statins, beta-blockers, and angiotensin-converting enzyme inhibitors, have been shown to effectively reduce the risk of cardiovascular events. However, these therapies primarily focus on managing symptoms and secondary complications of CAD, rather than addressing the underlying metabolic and inflammatory processes driving the disease [6,7]. Recently, the therapeutic landscape has shifted with the advent of pharmacological agents that target the underlying pathophysiology of CAD, particularly in patients with T2DM. Sodium-glucose cotransporter-2 inhibitors (SGLT2i) and glucagon-like peptide-1 receptor agonists (GLP-1 RAs) have emerged as crucial agents not only for glycemic control but also for their broad cardiovascular protective effects, particularly in individuals with CAD [8,9]. This review explores the mechanistic underpinnings of these agents, the clinical data supporting their cardiovascular benefits, and their potential role in combination therapy for patients with CAD.

## 2. Mechanistic Basis of Cardiovascular Protection

**SGLT2i.** Sodium-glucose cotransporter-2 inhibitors exert their primary effect by inhibiting the SGLT2 protein in the renal proximal tubules, which is responsible for glucose reabsorption [9]. This inhibition promotes urinary glucose excretion, thereby lowering plasma glucose levels. However, the cardiovascular benefits of SGLT2i extend well beyond glycemic control and involve multiple interrelated mechanisms (Table 1). One key mechanism is the modulation of myocardial sodium and calcium homeostasis. SGLT2i inhibits the myocardial sodium-hydrogen exchanger (NHE1), reducing intracellular sodium and calcium accumulation—particularly important during ischemia–reperfusion injury, such as in AMI [10,11]. By preserving calcium balance in cardiomyocytes, SGLT2i protect mitochondrial function, reduce oxidative stress, and limit apoptosis [10,11]. This leads to reduced infarct size and preservation of myocardial contractility following AMI. Additionally, these agents reduce myocardial fibrosis and improve left ventricular function, both critical for long-term recovery and heart failure prevention. SGLT2i also exerts anti-inflammatory effects [12,13]. Through activation of AMP-activated protein kinase—a central regulator of cellular energy metabolism—they suppress pro-inflammatory signaling pathways such as NF-κB [13,14]. This helps mitigate chronic vascular inflammation, a key driver of atherosclerosis and plaque instability. Moreover, by reducing reactive oxygen species production, SGLT2i lowers oxidative stress, protects endothelial cells, and reduces LDL oxidation—important factors in atherosclerotic plaque development. Improved endothelial function, including enhanced nitric oxide bioavailability and reduced arterial stiffness, contributes to better vascular tone and reduced ischemic risk [13,14]. Hemodynamic benefits also play a role: by promoting osmotic diuresis and natriuresis, SGLT2i lowers blood volume and preload, reducing myocardial oxygen demand [9,14,15]. These changes help optimize the balance between oxygen supply and demand—especially beneficial in CAD and heart failure. Furthermore, decreased afterload and improved ventricular remodeling contribute to reduced heart failure risk and progression in patients with CAD and AMI.

**GLP-1 Receptor Agonists.** Glucagon-like peptide-1 RAs mimic the effects of endogenous GLP-1, a peptide hormone central to glucose metabolism [16]. These agents promote glucose-dependent insulin secretion, inhibit glucagon release, slow gastric emptying, and enhance satiety—contributing to effective glycemic control [16]. However, the cardiovascular benefits of GLP-1 RAs extend beyond glucose regulation, involving several mechanisms including improved endothelial function, reduced oxidative stress and inflammation, and stabilization of atherosclerotic plaques [17,18] (Table 1). A key mechanism underlying their cardiovascular protection is the upregulation of endothelial nitric oxide synthase, leading to increased production of nitric oxide—a potent vasodilator that improves blood flow, reduces shear stress, and protects the endothelium. This is particularly relevant in CAD, where endothelial dysfunction plays a central role in disease progression [4,5]. By enhancing endothelial function, GLP-1 RAs help reduce the risk of plaque rupture and adverse cardiovascular events [19,20]. GLP-1 RAs also exert robust anti-inflammatory effects that contribute to plaque stability. They reduce the expression of pro-inflammatory cytokines such as interleukin-6 (IL-6) and tumor necrosis factor-alpha, both of which are involved in atherogenesis and plaque destabilization [13,21]. Furthermore, these agents inhibit macrophage infiltration and suppress matrix metalloproteinase activity—two critical processes involved in fibrous cap degradation and thrombus formation [13,21]. Preclinical studies suggest that GLP-1 RAs enhance plaque stability by increasing fibrous cap thickness and reducing necrotic core size, providing direct structural protection against rupture [7,9]. In addition, GLP-1 RAs improve myocardial glucose uptake, particularly during ischemia–reperfusion events. By enhancing mitochondrial function and preserving ATP production, they help protect cardiomyocytes from ischemic damage [18,21]. This leads to reduced infarct size and improved outcomes in AMI—a crucial benefit in preserving long-term myocardial function.

## 3. Clinical Evidence in CAD

**SGLT2 Inhibitors.** The cardiovascular benefits of SGLT2i have been firmly established in multiple large randomized controlled trials involving patients with T2DM and CAD [22] (Table 2). The EMPAgliflozin REGistration for cardiovascular OUTCOME event trial in Type 2 Diabetes Mellitus (EMPA-REG OUTCOME) trial (7020 patients) demonstrated a 38% reduction in cardiovascular death, 35% in heart failure hospitalizations, and 14% in major adverse cardiovascular events (MACE) with empagliflozin [9,23]. The Canagliflozin Cardiovascular Assessment Study (CANVAS) program (10,142 patients) showed a similar ~14% MACE reduction and improved heart failure outcomes with canagliflozin [24]. The Dapagliflozin Effect on CardiovascuLAR Events (DECLARE) TIMI 58 trial reinforced these findings, and dapagliflozin significantly reduced cardiovascular death and heart failure hospitalizations, confirming a broader therapeutic scope [25]. Differently, the Cardiovascular Outcomes Following Ertugliflozin Treatment in Patients with Type 2 Diabetes Mellitus and Atherosclerotic Cardiovascular Disease (VERTIS CV), demonstrated that ertugliflozin was non-inferior (but not superior) to placebo for the primary composite outcome of MACE, but confirmed class effect in reducing the risk of hospitalization for heart failure, with a 30% reduction [26,27]. More recently, trials have focused on patients in the post-AMI setting. The EMPAgliflozin for the Prevention of Chronic Heart Failure and morTality after an Acute Myocardial Infarction (EMPACT MI) trial started empagliflozin within 14 days post-AMI; while it did not significantly reduce the primary composite endpoint of death or heart failure hospitalization, it lowered the risk of heart failure hospitalization [28,29]. The dapagliflozin in patients with myocardial infarction (DAPA MI) trial enrolled approximately 4000 post-AMI, non-DM patients and demonstrated superiority of dapagliflozin in improving cardiometabolic composite outcomes [30]. These results support the use of SGLT2i in improving long-term risk profiles after AMI, especially through cardiometabolic mechanisms, though definitive mortality benefits require further confirmation (Table 2). Notably, the use of sotagliflozin, a dual SGLT1/SGLT2 inhibitor, in high-risk patients has been associated with ~23% reduction in composite risk of myocardial infarction, stroke, and cardiovascular death [31,32]. Collectively, these trials underscore the dual benefit of SGLT2i: while initially developed for glycemic control, they provide substantial cardiovascular protection—particularly in patients with CAD and in the critical period following an AMI.

**GLP-1 Receptor Agonists.** Glucagon-like peptide-1 receptor agonists have demonstrated substantial cardiovascular benefits in patients with T2DM and those with established CAD (Table 3). The Liraglutide Effect and Action in Diabetes: Evaluation of Cardiovascular Outcome Results (LEADER) trial demonstrated a 13% reduction in MACE and a 22% decrease in cardiovascular death with liraglutide in patients with DM and high cardiovascular risk [8]. Similarly, in the trial to Evaluate Cardiovascular and Other Long-term Outcomes with Semaglutide (SUSTAIN-6), subcutaneous semaglutide led to a 26% reduction in MACE and a 39% decrease in non-fatal stroke, further supporting the cardiovascular efficacy of GLP-1 RAs [33]. Oral formulation of semaglutide also recently showed in the Semaglutide Outcomes Study (SOUL) a 14% reduction in MACE [34]. The Researching Cardiovascular Events with a Weekly INcretin in Diabetes (REWIND) trial, which included a broader T2DM population with lower cardiovascular risk, found that dulaglutide reduced MACE by 12%, highlighting the benefits of GLP-1RAs across a wider spectrum of patients [35]. These agents contribute to plaque stabilization, reduce the risk of AMI, and improve outcomes in patients with CAD through mechanisms including enhanced endothelial function and anti-inflammatory effects [36]. In the context of AMI, clinical studies support the cardioprotective role of GLP-1RAs. A meta-analysis of randomized trials and cohort studies showed that these agents reduce infarct size by approximately 5.29 g and improve left ventricular ejection fraction by 2.46%—both critical outcomes in post-AMI recovery [37] (Table 3). Additionally, observational studies indicate that GLP-1 RAs use before ST-elevation myocardial infarction (STEMI) is associated with a 40% reduction in long-term mortality [38]. These findings are especially relevant in the post-AMI setting, where mitigating myocardial injury and preserving cardiac function are key to preventing heart failure and subsequent cardiovascular complications. By stabilizing atherosclerotic plaques, improving endothelial function, and reducing inflammation, GLP-1RAs play a significant role in improving cardiovascular outcomes in patients with CAD. Finally, the Semaglutide Effects on Heart Disease and Stroke in Patients With Overweight or Obesity (SELECT) trial highlighted that weekly semaglutide reduced MACE in obese patients without DM but with established cardiovascular disease [39]. These findings further demonstrate that the ability of GLP-1 RAs to stabilize plaques, improve endothelial function, and reduce inflammation contributes significantly to improved cardiovascular outcomes.

## 4. Renal Protection by SGLT2 Inhibitors and GLP-1 Receptor Agonists in CAD

Renal protection is a critical component of the therapeutic benefits provided by SGLT2 and GLP-1 RAs, particularly in patients with CAD and Type 2 Diabetes Mellitus (T2DM), where chronic kidney disease (CKD) is highly prevalent and significantly worsens prognosis [25]. The coexistence of CAD, T2DM, and CKD defines a high-risk clinical phenotype associated with increased cardiovascular morbidity and mortality, accelerated renal function decline, and higher rates of hospitalization and adverse outcomes. In this context, preserving renal function is not only essential to improving long-term quality of life and reducing dialysis burden, but is also strongly linked to improved cardiovascular outcomes. Both SGLT2i and GLP-1 RAs have emerged as cornerstone therapies capable of slowing CKD progression and reducing renal complications through distinct but potentially complementary mechanisms.

SGLT2 inhibitors have emerged as potent renoprotective agents. Their primary mechanisms include reducing glomerular hyperfiltration, lowering intraglomerular pressure, and decreasing albuminuria—all of which contribute to slowing CKD progression [40]. Large clinical trials such as the EMPA-REG OUTCOME, CANVAS Program, and VERTIS CV revealed that these agents lowered the risk of progression to end-stage renal disease in patients with DM. For instance, in the EMPA-REG OUTCOME trial, empagliflozin was associated with a 39% reduction in the risk of kidney disease progression, including a decrease in the need for renal replacement therapy [9]. The Study of Heart and Kidney Protection With Empagliflozin (EMPA-KIDNEY trial) demonstrated that empagliflozin not only reduces the risk of CKD progression and cardiovascular death but that these benefits can persist for up to 12 months after discontinuation [41]. A renal sub-analysis of the EMPACT-MI trial further showed that empagliflozin preserved kidney function in post-AMI patients, including those with impaired baseline renal function [30]. Similar renal protection was also found in patients with CKD randomized to dapagliflozin compared to placebo. The Dapagliflozin and Prevention of Adverse Outcomes in Chronic Kidney Disease (DAPA-CKD) trial showed that dapagliflozin reduced all-cause mortality and renal decline in CKD patients [42]. These effects are thought to result from a combination of hemodynamic mechanisms (e.g., reducing glomerular pressure) and non-hemodynamic actions, including anti-inflammatory, anti-fibrotic, and anti-oxidant properties.

GLP-1 RAs also offer renal benefits, though through different mechanisms. These agents have been shown to reduce albuminuria and, in some studies, stabilize or improve glomerular filtration rate (GFR) [9]. Their renal protective effects are largely attributed to anti-inflammatory, anti-oxidative, and anti-fibrotic actions, as well as improvements in endothelial function [9]. In the ELIXA (Evaluation of Cardiovascular Outcomes in Patients With Type 2 Diabetes After Acute Coronary Syndrome During Treatment With Lixisenatide) trial, lixisenatide was associated with slowed progression of albuminuria in patients with T2DM and established cardiovascular disease [43]. While SGLT2i seems to provide stronger hemodynamic benefits, GLP-1 RAs complement these effects metabolically and at the cellular level.

Emerging evidence suggests that combining SGLT2i with GLP-1RAs may offer even greater renal protection by targeting both the metabolic and hemodynamic contributors to CKD. Their action—improving glucose homeostasis, mitigating inflammation, and enhancing endothelial function—could synergistically slow CKD progression and reduce the burden of renal and cardiovascular complications in patients with CAD and DM. However, further studies are needed to fully define the extent and durability of their combined renal benefits.

Preliminary data from retrospective studies suggest that SGLT2i may help prevent contrast-induced acute kidney injury (CI-AKI)—a serious complication with poor prognosis, particularly in patients hospitalized with AMI undergoing urgent or primary percutaneous coronary intervention, where implementing prophylactic measures is often challenging [44]. Experimental evidence supports this hypothesis. SGLT2i have been shown to restore intracellular hypoxic conditions by reducing the oxygen consumption rate of tubular cells in diabetic kidneys [45,46]. Furthermore, a recent animal study suggested that hypoxic injury is a key pathological mechanism underlying CI-AKI. In this study, dapagliflozin appeared to exert a protective effect, at least in part, by suppressing the HIF-1α/HE4/NF-κB signaling pathway—a transcriptional cascade activated under hypoxic conditions [47]. Consistent with this, some retrospective studies have reported a lower incidence of CI-AKI in AMI patients with DM who were chronically treated with an SGLT2 inhibitor. For example, in a cohort of 295 STEMI patients with DM undergoing primary percutaneous coronary intervention, CI-AKI occurred less frequently among those on SGLT2i therapy (9% vs. 18%), with an adjusted odds ratio of 0.86 (95% CI: 0.76–0.98) [48]. Similar findings emerged from the Cardioprotective Effect of SGLT2-I in Diabetic Patients With AMI (SGLT-I AMI PROTECT) registry, which included 646 DM patients (111 SGLT2i users and 535 non-users). The incidence of CI-AKI was significantly lower in SGLT2i users (5.4% vs. 13.1%; *p* = 0.02) [49]. However, these findings should be interpreted with caution. Notably, the existing studies have included only patients with DM, leaving it unclear whether similar renoprotective effects extend to non-DM patients with AMI. Furthermore, data on the pharmacodynamics underlying this potential benefit are lacking. In the reported studies, patients had been on SGLT2i for at least six months. It remains unknown whether similar benefits would be observed if SGLT2i were initiated shortly before contrast exposure—such as 30 min or even 24 h in advance. Importantly, major randomized trials of SGLT2i have consistently demonstrated an initial decline in renal function following treatment initiation, despite clear long-term renal protective effects. Whether acute administration could still offer benefit remains an open question. These uncertainties underscore the need for further investigation through well-designed, prospective, randomized trials. In conclusion, while SGLT2i show promise as adjunctive agents in the prevention of CI-AKI—potentially expanding their utility beyond current indications—further investigation is needed. Randomized controlled trials with larger, more diverse cohorts, including non-DM AMI patients, are essential to validate these preliminary observations. As research advances, SGLT2i may emerge as a valuable strategy to protect renal function in high-risk cardiac patients undergoing percutaneous coronary intervention.

## 5. Exploring the Potential Synergy in Combination Therapy

Given their distinct yet complementary mechanisms of action, there is growing interest in the potential synergistic effects of combining SGLT2i and GLP-1RA for the management of CAD (Central Illustration). SGLT2i primarily confer cardiovascular protection through metabolic, hemodynamic, and anti-inflammatory mechanisms, whereas GLP-1 RA offers benefits by improving endothelial function, promoting plaque stabilization, and providing direct myocardial protection (Table 4). Preclinical and preliminary data suggest that combining these therapies may lead to greater reductions in MACE, hospitalizations for heart failure, and AMI recurrence than monotherapy alone [50,51]. Furthermore, their complementary effects on metabolic control, vascular tone, and inflammation may allow for a more holistic approach to addressing multiple aspects of CAD pathophysiology [52]. Observational studies have reported that dual therapy may lead to greater reductions in MACE, as well as improved outcomes such as reduced rates of heart failure hospitalization and recurrent AMI. The complementary effects of these agents on metabolic regulation, vascular tone, and inflammatory pathways support a more comprehensive strategy for addressing the multifactorial pathophysiology of CAD. This therapeutic approach appears particularly promising in patients with both T2DM and CAD, where targeting both metabolic dysfunction and vascular pathology may enhance clinical outcomes. However, despite encouraging early evidence, robust data from large-scale, randomized controlled trials are needed to definitively assess the safety, efficacy, and long-term benefits of combining SGLT2i and GLP-1RA in patients with CAD, especially those with a prior history of AMI.

Finally, although both SGLT2 inhibitors and GLP-1 receptor agonists provide cardiovascular benefits in patients with CAD, differences exist in the magnitude, timing, and context of their effects. SGLT2i demonstrate a faster onset of cardiovascular benefit, with reductions in heart failure hospitalizations and cardiovascular death observed within weeks in trials [23,24,25,26,27,28,29,30,31,32]. This rapid benefit is thought to result from hemodynamic effects, including diuresis, natriuresis, and afterload reduction. Conversely, GLP-1 RAs exhibit more gradual effects, often requiring several months to show significant cardiovascular outcomes, as observed in the LEADER [8] and REWIND [35] trials, primarily driven by anti-atherosclerotic, anti-inflammatory, and metabolic actions. In acute CAD settings, especially post-AMI, SGLT2i appear particularly promising due to their early myocardial and renal protection, although formal evidence in non-diabetic populations is still emerging. GLP-1 RAs may offer greater benefit in chronic CAD, with data supporting long-term plaque stabilization.

## 6. Conclusions

The advent of SGLT2i and GLP-1RA has ushered in a paradigm shift in the management of CAD and AMI, particularly among patients with DM. These agents provide cardiovascular benefits that extend beyond glycemic control, exerting protective effects through mechanisms such as improved endothelial function, reduced systemic inflammation, enhanced myocardial preservation, and stabilization of atherosclerotic plaques.

Robust clinical evidence from multiple large-scale trials has demonstrated the efficacy of both drug classes in reducing MACE, heart failure hospitalizations, and cardiovascular mortality in patients with established CAD. Importantly, emerging data suggest that combination therapy with SGLT2i and GLP-1 RA may offer additive or even synergistic benefits compared to monotherapy, owing to their complementary mechanisms of action. This dual therapeutic approach holds particular promise in addressing the complex and multifactorial pathophysiology of CAD, offering a more comprehensive strategy for cardiovascular risk reduction. As ongoing research continues to clarify their roles, the use of these agents is likely to expand beyond diabetic populations, potentially transforming the treatment landscape for both diabetic and non-diabetic patients with CAD.

## Figures and Tables

**Table 1:** Comparative Overview of SGLT2 Inhibitors and GLP-1 Receptor Agonists in CAD.

Key Aspect	SGLT2 Inhibitors	GLP-1 Receptor Agonists
Primary use	Glycemic control; ↓ cardiovascular and renal events	Glycemic control; ↓ cardiovascular and renal events; ↓ body weight
Mechanisms of action	Inhibit renal glucose reabsorption; ↓ intracellular Na^+^/Ca^2+^; activate AMPK; ↓ oxidative stress	Mimic GLP-1; ↑ insulin, ↓ glucagon; modulate inflammation, appetite, and atherosclerotic plaque biology
Myocardial protection	↓ infarct size via NHE1 inhibition; preserve mitochondrial function; ↑ calcium homeostasis; ↓ apoptosis	↑ myocardial glucose uptake; ↑ ATP production; ↓ ischemic damage; ↓ apoptosis
Endothelial function	↑ NO bioavailability; ↓ arterial stiffness	↑ eNOS expression; ↑ NO production; improved endothelial resilience and barrier function
Anti-inflammatory effects	↓ NF-κB, IL-6, TNF-α; ↓ ROS; ↑ AMPK; ↓ macrophage activation	↓ IL-6, TNF-α; ↓ macrophage infiltration; ↓ MMP activity; ↑ plaque stabilization
Plaque stabilization	↓ LDL oxidation; ↓ vascular inflammation; ↑ endothelial integrity	↑ fibrous cap thickness; ↓ necrotic core; ↑ collagen content
Hemodynamic effects	↓ preload and afterload; natriuresis and osmotic diuresis	Minimal direct effect; metabolic improvements indirectly reduce hemodynamic burden
Renal protection	↓ albuminuria; preserve eGFR; protect against CKD progression (e.g., EMPA-KIDNEY, DAPA-CKD)	↓ albuminuria; anti-inflammatory and anti-fibrotic renal effects; slower CKD progression (e.g., ELIXA)
Effectiveness in AMI	Post-AMI benefit: improved remodeling and kidney preservation (e.g., EMPACT-MI); ↓ CI-AKI risk	Effective in AMI settings (e.g., ELIXA); potential additive benefit in cardiac recovery
Synergistic Potential	Enhanced cardiometabolic protection when combined with GLP-1 RAs	Complementary with SGLT2i: additive effects on metabolic, endothelial, and anti-inflammatory pathways

Abbreviations: AMI—Acute Myocardial Infarction; CAD—Coronary Artery Disease; AMPK—AMP-Activated Protein Kinase; ATP—Adenosine Triphosphate; CI-AKI—Contrast-Induced Acute Kidney Injury; CKD—Chronic Kidney Disease; eGFR—Estimated Glomerular Filtration Rate; eNOS—Endothelial Nitric Oxide Synthase; GLP-1 RA—Glucagon-Like Peptide-1 Receptor Agonist; IL-6—Interleukin-6; MMP—Matrix Metalloproteinase; Na^+^/Ca^2+^—Sodium/Calcium ions; NF-κB—Nuclear Factor kappa B; NO—Nitric Oxide; ROS—Reactive Oxygen Species; SGLT2i—Sodium–Glucose Cotransporter 2 Inhibitor; TNF-α—Tumor Necrosis Factor-alpha.

**Table 2:** Clinical Evidence of Cardiovascular Benefits in CAD of SGLT2 Inhibitors.

Study (Intervention)	Year	SGLT2 Inhibitors
EMPA-REG OUTCOME (Empagliflozin)	2015	↓ 38% CV death; ↓ 35% HF hospitalization; ↓ 14% MACE
CANVAS Program (Canagliflozin)	2017	↓ 14% MACE; ↓ HF hospitalization
DECLARE–TIMI 58 (Dapagliflozin)	2019	↓ CV death and HF hospitalization. Benefits extend to non-diabetic populations
VERTIS CV (ertugliflozin)	2020	No effect on MACE; ↓ 30% HF hospitalization
DAPA-MI (Dapagliflozin)	2023	No effect on composite CV death + HF hospitalization. Improved cardiometabolic outcomes (win-ratio)
EMPACT-MI (Empagliflozin)	2024	Neutral on primary composite. ↓ First and total HF hospitalizations

CV death = cardiovascular death, HF = heart failure, MACE = major adverse cardiovascular events.

**Table 3:** Clinical Evidence of Cardiovascular Benefits in CAD of GLP-1 Receptor Agonists.

Study (Intervention)	Year	GLP-1 Receptor Agonists
LEADER (Liraglutide)	2016	↓ 13% MACE ↓ 22% CV death
SUSTAIN-6 (Semaglutide)	2016	↓ 26% MACE ↓ 39% non-fatal stroke
SOUL (oral semaglutide	2025	↓ 14% MACE
REWIND (Dulaglutide)	2019	↓ 12% MACE (in a lower-risk T2DM population)
SELECT (Semaglutide)	2023	↓ MACE in overweight/obese non-diabetic individuals with established CVD

CV death = cardiovascular death, HF = heart failure, MACE = major adverse cardiovascular events.

**Table 4:** Synergistic Potential of SGLT2 Inhibitors and GLP-1 Receptor Agonists.

Combination Therapy	Potential Benefits	Challenges/Considerations
Synergistic Mechanisms	Complementary action on glycemia, weight, inflammation, endothelial function, and cardiac metabolism	Further randomized controlled trials are necessary to validate long-term outcomes
Targeted Patient Population	Patients with T2DM and CAD Post-AMI patients Possibly obese or non-diabetic individuals with CVD CKD patients, with or without DM, with or without obesity	- Need for large-scale randomized controlled trials - Higher costs
Potential Outcomes	↓ MACE ↓ HF hospitalization ↑ myocardial salvage index (observational and meta-analytic data) ↓ albuminuria, preserved eGFR, ↓ CKD progression	- Combined effects on multiple aspects of CAD pathophysiology
Effect on Long-term Mortality	Promising reduction in long-term cardiovascular mortality	- Lack of long-term evidence for combination therapy

AMI: acute myocardial infarction; CAD: coronary artery disease; CKD: chronic kidney disease; CVD: cardiovascular disease; eGFR: estimated glomerular filtration rate; GI: gastrointestinal; GLP-1 RAs: Glucagon-Like Peptide-1 Receptor Agonists; HF: heart failure; MACE: major adverse cardiovascular events; SGLT2 inhibitors: sodium–glucose cotransporter-2 inhibitors; T2DM: type 2 diabetes mellitus.

## Data Availability

Not applicable.

## References

[1] Libby P. (2002). Inflammation in atherosclerosis. Nature.

[2] Benjamin E.J., Muntner P., Alonso A., Bittencourt M.S., Callaway C.W., Carson A.P., Chamberlain A.M., Chang A.R., Cheng S., Das S.R. (2019). Heart Disease and Stroke Statistics-2019 Update: A Report From the American Heart Association. Circulation.

[3] Thygesen K., Alpert J.S., Jaffe A.S., Chaitman B.R., Bax J.J., Morrow D.A., White H.D., Executive Group on behalf of the Joint European Society of Cardiology (ESC)/American College of Cardiology (ACC)/American Heart Association (AHA)/World Heart Federation (WHF) Task Force for the Universal Definition of Myocardial Infarction (2018). Fourth Universal Definition of Myocardial Infarction. Circulation.

[4] Beckman J.A., Creager M.A., Libby P. (2002). Diabetes and atherosclerosis: Epidemiology, pathophysiology, and management. JAMA.

[5] Donath M.Y., Shoelson S.E. (2011). Type 2 diabetes as an inflammatory disease. Nat. Rev. Immunol..

[6] Stone N.J., Robinson J.G., Lichtenstein A.H., Bairey Merz C.N., Blum C.B., Eckel R.H., Goldberg A.C., Gordon D., Levy D., Lloyd-Jones D.M. (2014). 2013 ACC/AHA guideline on the treatment of blood cholesterol to reduce atherosclerotic cardiovascular risk in adults: A report of the American College of Cardiology/American Heart Association Task Force on Practice Guidelines. J. Am. Coll. Cardiol..

[7] Yusuf S., Hawken S., Ounpuu S., Dans T., Avezum A., Lanas F., McQueen M., Budaj A., Pais P., Varigos J. (2004). Effect of potentially modifiable risk factors associated with myocardial infarction in 52 countries (the INTERHEART study): Case-control study. Lancet.

[8] Marso S.P., Daniels G.H., Brown-Frandsen K., Kristensen P., Mann J.F., Nauck M.A., Nissen S.E., Pocock S., Poulter N.R., Ravn L.S. (2016). Liraglutide and Cardiovascular Outcomes in Type 2 Diabetes. N. Engl. J. Med..

[9] Zinman B., Wanner C., Lachin J.M., Fitchett D., Bluhmki E., Hantel S., Mattheus M., Devins T., Johansen O.E., Woerle H.J. (2015). Empagliflozin, Cardiovascular Outcomes, and Mortality in Type 2 Diabetes. N. Engl. J. Med..

[10] Packer M. (2017). Activation and Inhibition of Sodium-Hydrogen Exchanger Is a Mechanism That Links the Pathophysiology and Treatment of Diabetes Mellitus With That of Heart Failure. Circulation.

[11] Yurista S.R., Sillje H.H.W., Oberdorf-Maass S.U., Schouten E.M., Pavez Giani M.G., Hillebrands J.L., van Veldhuisen D.J., de Boer R.A., Westenbrink B.D. (2019). Sodium-glucose co-transporter 2 inhibition with empagliflozin improves cardiac function in non-diabetic rats with left ventricular dysfunction after myocardial infarction. Eur. J. Heart Fail..

[12] Shi Y., Evans J.E., Rock K.L. (2003). Molecular identification of a danger signal that alerts the immune system to dying cells. Nature.

[13] Kim S.R., Lee S.G., Kim S.H., Kim J.H., Choi E., Cho W., Flores E., Garcia-Ropero A., Sanz J., Hajjar R.J. (2020). SGLT2 inhibition modulates NLRP3 inflammasome activity via ketones and insulin in diabetes with cardiovascular disease. Nat. Commun..

[14] Santos-Gallego C.G., Requena-Ibanez J.A., San Antonio R., Ishikawa K., Watanabe S., Picatoste B., Flores E., Garcia-Ropero A., Sanz J., Hajjar R.J. (2019). Empagliflozin Ameliorates Adverse Left Ventricular Remodeling in Nondiabetic Heart Failure by Enhancing Myocardial Energetics. J. Am. Coll. Cardiol..

[15] Verma S., McMurray J.J.V. (2018). SGLT2 inhibitors and mechanisms of cardiovascular benefit: A state-of-the-art review. Diabetologia.

[16] Drucker D.J. (2018). Mechanisms of Action and Therapeutic Application of Glucagon-like Peptide-1. Cell Metab..

[17] Koniari I., Velissaris D., Kounis N.G., Koufou E., Artopoulou E., de Gregorio C., Mplani V., Paraskevas T., Tsigkas G., Hung M.-Y. (2022). Anti-Diabetic Therapy, Heart Failure and Oxidative Stress: An Update. J Clin Med..

[18] Kataoka Y., Kitahara S., Funabashi S., Makino H., Matsubara M., Matsuo M., Omura-Ohata Y., Koezuka R., Tochiya M., Tamanaha T. (2024). Glucagon-like Peptide-1 analogues and delipidation of coronary atheroma in statin-treated type 2 diabetic patients with coronary artery disease: The prespecified sub-analysis of the OPTIMAL randomized clinical trial. Atheroscler. Plus..

[19] Menghini R., Casagrande V., Rizza S., Federici M. (2023). GLP-1RAs and cardiovascular disease: Is the endothelium a relevant platform?. Acta Diabetol..

[20] Burgmaier M., Liberman A., Mollmann J., Kahles F., Reith S., Lebherz C., Marx N., Lehrke M. (2013). Glucagon-like peptide-1 (GLP-1) and its split products GLP-1(9-37) and GLP-1(28-37) stabilize atherosclerotic lesions in apoe^−/−^ mice. Atherosclerosis.

[21] Skrobucha A., Pindlowski P., Krajewska N., Grabowski M., Jonik S. (2024). Anti-inflammatory effects of glucagon-like peptide-1 (GLP-1) in coronary artery disease: A comprehensive review. Front. Cardiovasc. Med..

[22] McGuire D.K., Shih W.J., Cosentino F., Charbonnel B., Cherney D.Z.I., Dagogo-Jack S., Pratley R., Greenberg M., Wang S., Huyck S. (2021). Association of SGLT2 Inhibitors With Cardiovascular and Kidney Outcomes in Patients With Type 2 Diabetes: A Meta-analysis. JAMA Cardiol..

[23] Fitchett D., Zinman B., Wanner C., Lachin J.M., Hantel S., Salsali A., Johansen O.E., Woerle H.J., Broedl U.C., Inzucchi S.E. (2016). Heart failure outcomes with empagliflozin in patients with type 2 diabetes at high cardiovascular risk: Results of the EMPA-REG OUTCOME(R) trial. Eur. Heart J..

[24] Neal B., Perkovic V., Matthews D.R. (2017). Canagliflozin and Cardiovascular and Renal Events in Type 2 Diabetes. N. Engl. J. Med..

[25] Wiviott S.D., Raz I., Bonaca M.P., Mosenzon O., Kato E.T., Cahn A., Silverman M.G., Zelniker T.A., Kuder J.F., Murphy S.A. (2019). Dapagliflozin and Cardiovascular Outcomes in Type 2 Diabetes. N. Engl. J. Med..

[26] Cosentino F., Cannon C.P., Cherney D.Z.I., Masiukiewicz U., Pratley R., Dagogo-Jack S., Frederich R., Charbonnel B., Mancuso J., Shih W.J. (2020). Efficacy of Ertugliflozin on Heart Failure-Related Events in Patients With Type 2 Diabetes Mellitus and Established Atherosclerotic Cardiovascular Disease: Results of the VERTIS CV Trial. Circulation.

[27] Cannon C.P., Pratley R., Dagogo-Jack S., Mancuso J., Huyck S., Masiukiewicz U., Charbonnel B., Frederich R., Gallo S., Cosentino F. (2020). Cardiovascular Outcomes with Ertugliflozin in Type 2 Diabetes. N. Engl. J. Med..

[28] Butler J., Jones W.S., Udell J.A., Anker S.D., Petrie M.C., Harrington J., Mattheus M., Zwiener I., Amir O., Bahit M.C. (2024). Empagliflozin after Acute Myocardial Infarction. N. Engl. J. Med..

[29] Hernandez A.F., Udell J.A., Jones W.S., Anker S.D., Petrie M.C., Harrington J., Mattheus M., Seide S., Zwiener I., Amir O. (2024). Effect of Empagliflozin on Heart Failure Outcomes After Acute Myocardial Infarction: Insights From the EMPACT-MI Trial. Circulation.

[30] James S., Erlinge D., Storey R.F., McGuire D.K., de Belder M., Eriksson N., Andersen K., Austin D., Arefalk G., Carrick D. (2024). Dapagliflozin in Myocardial Infarction without Diabetes or Heart Failure. NEJM Evid..

[31] Aggarwal R., Bhatt D.L., Szarek M., Cannon C.P., Leiter L.A., Inzucchi S.E., Lopes R.D., McGuire D.K., Lewis J.B., Riddle M.C. (2025). Effect of sotagliflozin on major adverse cardiovascular events: A prespecified secondary analysis of the SCORED randomised trial. Lancet Diabetes Endocrinol..

[32] Bhatt D.L., Szarek M., Steg P.G., Cannon C.P., Leiter L.A., McGuire D.K., Lewis J.B., Riddle M.C., Voors A.A., Metra M. (2021). Sotagliflozin in Patients with Diabetes and Recent Worsening Heart Failure. N. Engl. J. Med..

[33] Marso S.P., Bain S.C., Consoli A., Eliaschewitz F.G., Jodar E., Leiter L.A., Lingvay I., Rosenstock J., Seufert J., Warren M.L. (2016). Semaglutide and Cardiovascular Outcomes in Patients with Type 2 Diabetes. N. Engl. J. Med..

[34] McGuire D.K., Marx N., Mulvagh S.L., Deanfield J.E., Inzucchi S.E., Pop-Busui R., Mann J.F.E., Emerson S.S., Poulter N.R., Engelmann M.D.M. (2025). Oral Semaglutide and Cardiovascular Outcomes in High-Risk Type 2 Diabetes. N. Engl. J. Med..

[35] Gerstein H.C., Colhoun H.M., Dagenais G.R., Diaz R., Lakshmanan M., Pais P., Probstfield J., Riesmeyer J.S., Riddle M.C., Rydén L. (2019). Dulaglutide and cardiovascular outcomes in type 2 diabetes (REWIND): A double-blind, randomised placebo-controlled trial. Lancet.

[36] Nauck M.A., Meier J.J., Cavender M.A., Abd El Aziz M., Drucker D.J. (2017). Cardiovascular Actions and Clinical Outcomes With Glucagon-Like Peptide-1 Receptor Agonists and Dipeptidyl Peptidase-4 Inhibitors. Circulation.

[37] Wong S.Y., Bin Lee A.R.Y., Sia A.H.J., Wo Y.J., Teo Y.N., Syn N.L., Ong C.-C., Teo L.L., Yeo T.-C., Poh K.-K. (2024). Effects of Glucagon-Like Peptide-1 Receptor Agonist (GLP-1RA) on Cardiac Structure and Function: A Systematic Review and Meta-Analysis of Randomized-Controlled Trials. Cardiovasc. Drugs Ther..

[38] Madsen J.M., Kjaer A.K., Lonborg J.T., Kober L., Glinge C., Jabbari R., Engstrøm T. (2025). Long-term prognostic impact of glucagon-like peptide-1 receptor agonist before ST-segment elevation myocardial infarction in patients with type 2 diabetes: A nationwide cohort study. Cardiovasc. Diabetol..

[39] Wilding J.P.H., Batterham R.L., Calanna S., Davies M., Van Gaal L.F., Lingvay I., McGowan B.M., Rosenstock J., Tran M.T., Wadden T.A. (2021). Once-Weekly Semaglutide in Adults with Overweight or Obesity. N. Engl. J. Med..

[40] Fioretto P., Zambon A., Rossato M., Busetto L., Vettor R. (2016). SGLT2 Inhibitors and the Diabetic Kidney. Diabetes Care.

[41] The E.-K.C.G., Herrington W.G., Staplin N., Wanner C., Green J.B., Hauske S.J., Emberson Gómez J.R. (2023). Empagliflozin in Patients with Chronic Kidney Disease. N. Engl. J. Med..

[42] Heerspink H.J.L., Stefansson B.V., Correa-Rotter R., Chertow G.M., Greene T., Hou F.F., Mann J.F., McMurray J.J., Lindberg M., Rossing P. (2020). Dapagliflozin in Patients with Chronic Kidney Disease. N. Engl. J. Med..

[43] Pfeffer M.A., Claggett B., Diaz R., Dickstein K., Gerstein H.C., Kober L.V., Lawson F.C., Ping L., Wei X., Lewis E.F. (2015). Lixisenatide in Patients with Type 2 Diabetes and Acute Coronary Syndrome. N. Engl. J. Med..

[44] Marenzi G., Cosentino N., Bartorelli A.L. (2015). Acute kidney injury in patients with acute coronary syndromes. Heart.

[45] Takiyama Y., Haneda M. (2014). Hypoxia in diabetic kidneys. Biomed. Res. Int..

[46] Blantz R.C. (2014). Phenotypic characteristics of diabetic kidney involvement. Kidney Int..

[47] Huang X., Guo X., Yan G., Zhang Y., Yao Y., Qiao Y., Wang D., Chen G., Zhang W., Tang C. (2022). Dapagliflozin Attenuates Contrast-induced Acute Kidney Injury by Regulating the HIF-1α/HE4/NF-κB Pathway. J. Cardiovasc. Pharmacol..

[48] Kültürsay B., Yılmaz C., Güven B., Mutlu D., Karagöz A. (2024). Potential renoprotective effect of SGLT2 inhibitors against contrast-induced AKI in diabetic STEMI patients undergoing primary PCI. Kardiol. Pol..

[49] Paolisso P., Bergamaschi L., Gragnano F., Gallinoro E., Cesaro A., Sardu C., Mileva N., Foà A., Armillotta M., Sansonetti A. (2023). Outcomes in diabetic patients treated with SGLT2-Inhibitors with acute myocardial infarction undergoing PCI: The SGLT2-I AMI PROTECT Registry. Pharmacol. Res..

[50] Trombara F., Cosentino N., Bonomi A., Ludergnani M., Poggio P., Gionti L., Baviera M., Colacioppo P., Roncaglioni M.C., Leoni O. (2023). Impact of chronic GLP-1 RA and SGLT-2I therapy on in-hospital outcome of diabetic patients with acute myocardial infarction. Cardiovasc. Diabetol..

[51] Marfella R., Prattichizzo F., Sardu C., Rambaldi P.F., Fumagalli C., Marfella L.V., La Grotta R., Frigé C., Pellegrini V., D’aNdrea D. (2024). GLP-1 receptor agonists-SGLT-2 inhibitors combination therapy and cardiovascular events after acute myocardial infarction: An observational study in patients with type 2 diabetes. Cardiovasc. Diabetol..

[52] Neuen B.L., Fletcher R.A., Heath L., Perkovic A., Vaduganathan M., Badve S.V., Tuttle K.R., Pratley R., Gerstein H.C., Perkovic V. (2024). Cardiovascular, Kidney, and Safety Outcomes With GLP-1 Receptor Agonists Alone and in Combination With SGLT2 Inhibitors in Type 2 Diabetes: A Systematic Review and Meta-Analysis. Circulation.

